# Can atypical antipsychotic drugs cause hepatotoxicity?

**DOI:** 10.1192/j.eurpsy.2021.411

**Published:** 2021-08-13

**Authors:** R. André, C. Sereijo, M. Abreu

**Affiliations:** 1 Psychiatry, Centro Hospital Lisboa Norte, Lisboa, Portugal; 2 Psiquiatria, Centro Hospitalar Universitario Lisboa Norte, Lisboa, Portugal

**Keywords:** Antipsychotics, hepatic damage, atypical antipsychotics, hepatotoxicity

## Abstract

**Introduction:**

Neuropsychiatric drugs account for 16% of drugs that can lead to hepatotoxicity and psychiatric patients can have multiple comorbidities that can increase the incidence of liver disorders such as alcoholism, drug abuse and polymedication. The continuous use of atypical antipsychotic drugs (AAD) has raised questions over their tolerability over endocrine, metabolic and cardiovascular systems. They are also associated with mild elevation of aminotransferases and occasionally cause idiosyncratic liver injury with varying phenotypes. Hepatotoxicity is defined based on biological parameters such as elevation of alkaline phosphatase enzyme, SGPT, SGOT and GGT or clinical abnormalities (jaundice and hepatitis).

**Objectives:**

This work reviewed the current available evidence on the hepatic damage produced by AAD.

**Methods:**

Non-systematic review of the literature with selection of scientific articles published in the past 10 years; by searching Pubmed and Medscape databases using the combination of MeSH descriptors. The following MeSH terms were used: atypical antipsychotic drugs; hepatotoxicity; hepatic; Olanzapine; Clozapine; Risperidone; Aripiprazol; Paliperidone.

**Results:**

Atypical Antipsychotic Drugs are generally well tolerated and hepatic alterations are in general very low or rare. The cases published were observed with Clozapine, Olanzapine and Risperidone. Atypical Antipsychotic drugs have a better profile than Chlorpromazine.

**Conclusions:**

In conclusion, the hepatic injury generally occurs within the first weeks of treatment and is usually reversible with drug withdrawal. Hepatic check-ups may be relevant, especially in the beginning of treatment.

**Disclosure:**

No significant relationships.

